# Bioinformatics challenges and perspectives when studying the effect of epigenetic modifications on alternative splicing

**DOI:** 10.1098/rstb.2017.0073

**Published:** 2018-04-23

**Authors:** Clare Pacini, Magdalena J. Koziol

**Affiliations:** 1 Wellcome Trust Cancer Research UK Gurdon Institute, University of Cambridge, Tennis Court Road, Cambridge, CB2 1QN, UK; 2 Department of Zoology, University of Cambridge, Downing Street, Cambridge, CB2 3EJ, UK

**Keywords:** epigenetics, splicing, RNA-SEQ, bioinformatics, modelling, networks

## Abstract

It is widely known that epigenetic modifications are important in regulating transcription, but several have also been reported in alternative splicing. The regulation of pre-mRNA splicing is important to explain proteomic diversity and the misregulation of splicing has been implicated in many diseases. Here, we give a brief overview of the role of epigenetics in alternative splicing and disease. We then discuss the bioinformatics methods that can be used to model interactions between epigenetic marks and regulators of splicing. These models can be used to identify alternative splicing and epigenetic changes across different phenotypes.

This article is part of a discussion meeting issue ‘Frontiers in epigenetic chemical biology’.

## 1. Introduction

Epigenetic modifications change the regulation of the genome without changing the DNA sequence. As such, our increased knowledge of epigenetics significantly improves our understanding of gene regulation and biological pathways that are regulated epigenetically. Epigenetic modifications can occur directly on DNA, and on histones that bind to DNA. For example, chemical modifications such as methylation, acetylation and phosphorylation are known to alter histone function. Each of these modifications is reversible, and this has increased the prospects of epigenetic markers as therapeutic drug targets [1,2]. In the case of eukaryotic DNA, mostly DNA methylation affecting cytosine residues (methylcytosine) has been extensively studied. Along with methylcytosine, other modified cytosine states have recently been identified, such as hydroxymethylcytosine, carboxylcytosine and formylcytosine [1]. Moreover, adenosine methylation has been found in the genome of various eukaryotes [3–7] and is not well understood. Further experimental and bioinformatics analysis of these modifications is required to understand their function. While DNA cytosine modifications are known to influence transcription, there is now also growing evidence for its role in the regulation of alternative splicing [8–10].

Alternative splicing of pre-mRNA from a single gene results in multiple isoforms that contribute greatly to the RNA and protein diversity in eukaryotes. Alternative splicing can include intron retention, alternative splice site selection and exon skipping. During splicing, the spliceosome, aided by splicing factors, recognizes and binds to specific sequences in the pre-mRNA. These sequences are at exon/intron boundaries and once the spliceosome binds to the sequence it excises the introns, which results in the mature mRNA [11]. The recruitment of the spliceosome is influenced by both *cis*-acting RNA elements and *trans*-acting splicing factors. *Cis*-acting RNA elements include enhancer or repressor sequences within the pre-mRNA that facilitate or inhibit the binding of the spliceosome. *Trans*-acting splicing factors can bind to the spliceosome or to bound or open *cis*-elements to direct binding of the spliceosome to the pre-mRNA and consequently alternative splicing.

The role of epigenetic modifications in alternative splicing has been extensively reviewed in detail [12–16]. Here, we only give a brief overview of the role of epigenetic modifications in alternative splicing. In this review, we focus on bioinformatics methods that have been developed to understand splicing regulation. This includes the regulatory relationships that govern splicing and differences in isoform expression relating to disease. We highlight the potential to integrate epigenetic markers in these analyses to elucidate the interaction between epigenetics and splicing. We provide some, though not exhaustive, examples on each topic while also directing the reader to comprehensive reviews.

## 2. Epigenetics and alternative splicing

In the classic view of splicing, transcription and splicing are two distinct processes, with transcription occurring first followed by splicing. However, splicing can occur co-transcriptionally [17]: introns can be spliced from pre-mRNA as the transcription of DNA occurs [18]. As a result, epigenetic modifications, such as histone and DNA modifications, are able to regulate splicing and transcription [8,13].

Chromatin structure can control splicing through access to and the recruitment of splicing factors. In yeast, the histone acetyltransferase Gcn5 was shown to interact with two U2 snRNP-encoding genes facilitating its recruitment to splice sites, which results in co-transcriptional splicing [19]. The compactness of chromatin structure also influences splicing through modifying the elongation rate of Polymerase II; a slower transcription rate has been shown to lead to differential splice site selection as weaker splice sites can be detected [20]. Weaker splice sites are *cis*-regulatory sequences at the exon–intron boundaries that are not as easily detected by the spliceosome. The subunit Brm of the chromatin remodeller, SWI/SNF, has been shown to regulate splicing. Brm causes an accumulation of RNA Polymerase II and pauses transcription. This seems to promote inclusion of exons with weaker splice sites, resulting in alternative splicing [21].

Histone modifications such as H3K36me3, which is significantly enriched in exons, have been implicated in the regulation of alternative splicing [22]. The H3K4me3 binding protein CHD1 was shown to facilitate splicing through the recruitment of the spliceosome component U2 snRNP. Knockdown of CHD1 resulted in reduced efficiency of splicing, while the combinatorial knockdown of CHD1 and H3K4me3 further reduced U2 snRNP recruitment [23].

**Figure 1.**
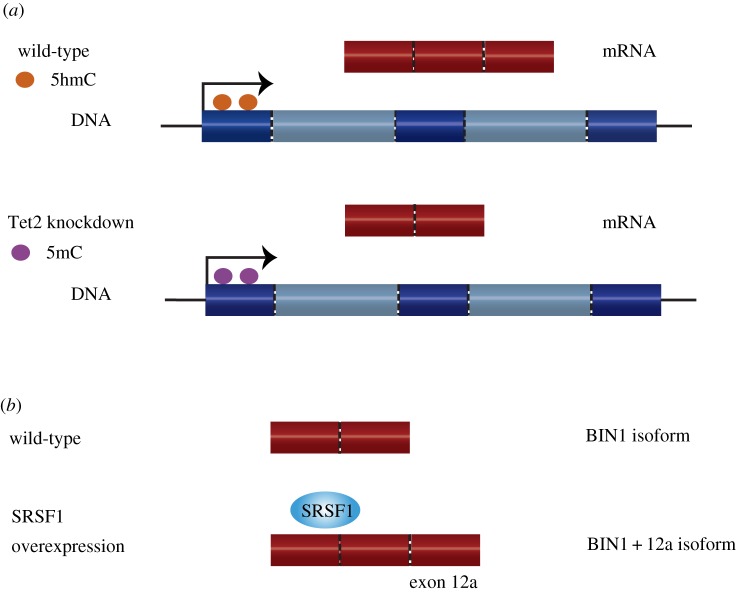
(*a*) Example of epigenetic markers influencing alternative splicing. Knockdown of the oxidase Tet2 has been shown to reduce levels of cytosine hydroxymethylation (5hmC) (orange circles). For some genomic regions with a reduction in 5hmC, a corresponding increase in cytosine methylation (5mC) was observed (blue circles). 5hmC was shown to be prevalent in exons, particularly at the boundaries, and the loss of 5hmC resulted in differential inclusion of exons. (*b*) The overexpression of splicing factor SRSF1 is known to cause differential splicing of BIN1. Overexpression of SRSF1 results in an alternative isoform BIN1+12a. This isoform is unable to bind the tumour repressor cMYC and therefore promotes tumorigenesis.

Finally, there is growing evidence of DNA modifications regulating splicing [13]. Increased DNA cytosine methylation in exons may regulate splicing by aiding the splicing machinery in the detection of exon/intron boundaries [24]. DNA cytosine methylation was significantly different between exons and introns, even when taking into consideration nucleosome and CpG density [25]. Moreover, knockdown of Tet2 decreases the level of cytosine hydroxymethylation and results in differential exon usage [26] (figure 1*a*). This further suggests a role of DNA modifications in alternative splicing.

Although we have focused here on examples of epigenetic modifications regulating splicing, splicing factors themselves can influence chromatin structure and histone modifications. This suggests a complex feedback model of regulation [11]. This complexity in the splicing process gives rise to multiple ways in which splicing can be misregulated and it is this misregulation that can lead to disease.

## 3. Alternative splicing and disease

It is known that certain mutations in splice sites, *cis*-regulatory elements, splicing factors and the spliceosome machinery can lead to disease. These diseases include chronic lymphocytic leukaemia, myelodysplastic syndrome and spinomuscular atrophy [12]. Furthermore, splicing factors have been shown to act as oncoproteins and tumour suppressors [27]. For example, the splicing factor SRSF1 is overexpressed in many tumours and found to influence processes leading to tumorigenesis [28]. Specific to alternative splicing, SRSF1 overexpression produces an alternative isoform of the protein BIN1 that is then unable to bind and repress the activity of the proto-oncogene cMYC [29] (figure 1*b*).

From an epigenetic perspective, the analysis of transcriptional networks of late onset Alzheimer's disease found that differentially expressed genes also had differential DNA cytosine methylation levels. This coincided with differential expression at the exon level and reduced splicing [30]. Another example that highlights the interplay between epigenetic markers, splicing and disease, is the protein MeCP2. Mutations of MeCP2 are known to cause neurodevelopmental disorders. MeCP2 can bind methylated cytosines and is involved in splicing. Exons bound by MeCP2 are enriched with specific epigenetic markers; histone modifications H3K4me3 and H3K36me3 are enriched in transcriptionally inactive and active exons, respectively. These regulated exons are involved in mRNA splicing and synaptic function within the brain. The model suggests that epigenetic markers facilitate splicing and the loss of this regulation can influence disease [31]. Despite these examples, the role of epigenetic modifications in alternative splicing is still not well understood. Therefore, identifying and analysing epigenetic drivers of alternative splicing may provide new insights into disease progression and treatment.

To elucidate the drivers of alternative splicing and disease progression, bioinformatics techniques need to be developed to model splicing regulation. To understand the effect of epigenetic modifications on splicing, observing and measuring the effects at the RNA transcript level are required [27]. To measure alternative splicing on a genome-wide scale, splice-aware or exon microarrays have previously been used [32]. RNA-seq is now the method of choice, as it has greater dynamic range and the ability to detect novel and known splice junctions. The analysis of RNA-seq data for alternative splicing requires computational models that are specifically designed for the analysis of differential transcripts. We discuss some of the methods that can be used with high-throughput data to model the relationships between splicing regulation, epigenetics and disease.

## 4. RNA-seq analysis of differential alternative splicing

**Figure 2.**
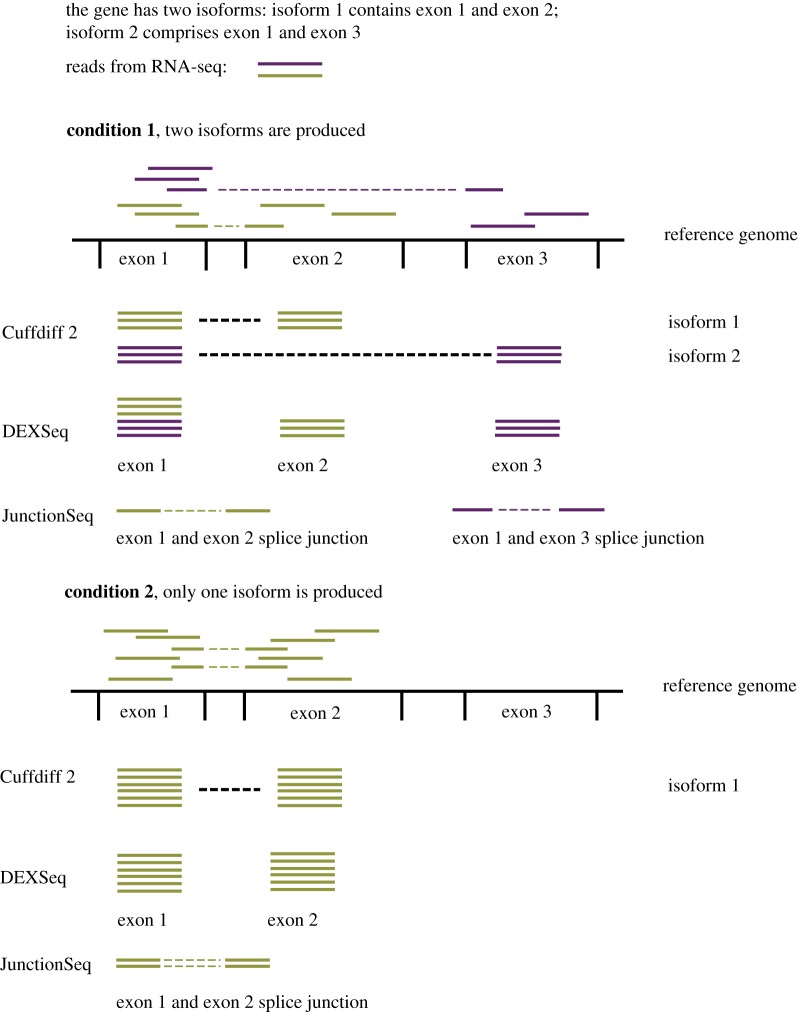
Sequencing reads from RNA-seq (shown as solid purple or green lines) are aligned to a reference genome. Three methods for analysing differential alternative splicing are shown: Cuffdiff 2, DEXSeq and JunctionSeq. The differences in how sequencing reads are assigned to give either isoform counts (Cuffdiff 2), exon counts (DEXSeq) or splice junction counts (JunctionSeq) are illustrated. For Cuffdiff 2, the difficulty is in correctly assigning the RNA-seq reads at exon 1 to isoform 1 (green reads) and isoform 2 (purple reads). For each method, the respective observed counts are used to detect differences in alternative splicing across two different conditions. The difference in these counts is shown; in the first condition, two isoforms of a gene are expressed and in the second condition only one isoform is expressed. For DEXSeq, no change in the expression of exon 1 will be detected. By contrast, JunctionSeq can detect a change in splicing involving exon 1 as there is an observed change in the number of reads at the exon 1 and exon 2 splice junctions.

There are multiple computational analysis tools designed for the analysis of alternative splicing using RNA-seq data. The most recent software packages estimate both biological and technical variation when testing for differential alternative splicing across conditions [33]. In differential alternative splicing, sequencing reads are first aligned to a reference genome. Given the aligned reads, one approach is to model directly observable exon counts (e.g. DEXSeq) and splice junction counts (e.g. JunctionSeq). The number of reads assigned to an exon or splice junction gives a measure of its expression. While DEXSeq models the observed exon counts, JunctionSeq uses both the observed exon counts and the counts at splice junctions. Therefore, JunctionSeq can identify differential splicing patterns of an exon even when the exon has consistent expression over all conditions. Alternatively, methods such as Cuffdiff 2, Tuxedo 2 and MISO assign reads to isoform transcripts and therefore model isoform as opposed to exon expression (figure 2). While this additional level of complexity in the modelling can be more error prone, Cuffdiff 2 has been shown to outperform differential exon usage analysis when the annotation is largely incomplete [34]. In selecting the correct tool for analysing splicing, it is important to consider whether replicates need to be modelled and the experimental conditions to be analysed. Two group (with replicates) and two sample (without replicates) comparisons can be done using tools such as DiffSplice and MATS, respectively. Experimental designs containing multiple groups or confounding factors would require a tool that can model design matrices such as JunctionSeq or Tuxedo 2. An overview of the available tools for differential alternative splicing analysis is shown in table 1. RNA-seq analysis can generate hypotheses of novel and known splice variants and it is advisable that any important alternative splicing results from RNA-seq analyses are verified. This can be done by either RT-PCR or sequencing capable of generating long full-length transcripts such as 454 Sequencing [44], SMRT sequencing [45] and Nanopore [35].

**Table 1.** Comparison of tools available for differential alternative splicing analysis. The tools calculate alternative splicing at different levels; isoform, exon or using splice junction counts. The tools differ in their ability to use biological replicates and the complexity of the experimental design. Tools that can only compare between two conditions are either two samples (where replicates are not used) or two groups (where replicates are used). Methods that use design matrices can incorporate more complex designs including multiple group comparison and confounding factors.

	biological replicates	model	experimental design	reference
JunctionSeq	yes	exon & junction	design matrix	[35]
Tuxedo 2	yes	isoform, exon & junction	design matrix	[36]
DEXSeq	yes	exon	design matrix	[37]
MATS	no	exon & junction	two sample	[38]
MISO	no	isoform	two sample	[39]
Cuffdiff 2	yes	isoform & exon	two groups	[40]
DSGseq	yes	exon	two groups	[41]
DiffSplice	yes	exon & junction	two groups	[42]
ARH-seq	yes	exon & junction	two sample	[43]

Results from differential alternative splicing analysis are usually interpreted via enrichment of biological function annotations provided by gene ontologies [36]. This enrichment analysis provides a good starting point to understanding results of differential alternative splicing. RNA-seq data give information on the observed changes in splicing at a transcript level. However, they provide little insight into the mechanisms that caused the observed changes in splicing. To understand interactions between elements, such as epigenetic marks, splicing factors and binding motifs, bioinformatics modelling is required. Bioinformatics models can integrate multiple data sources to reverse engineer and identify the effect of epigenetic landscapes on alternative splicing.

## 5. Bioinformatics modelling in epigenetics and splicing

In bioinformatics data analysis and modelling, there is a trade-off between the scale of the model and its complexity [37,38]. When only a few factors are being modelled, models can be built with detail. For tens of proteins, it is feasible to perturb them individually and in combination to resolve causal relationships between them [39]. By contrast, bioinformatics analysis of high-throughput data at an ‘omics’ scale is affected by the curse of dimensionality [40]. This refers to the comparatively large number of measured entities, such as genes, compared to the small number of replicates or experimental conditions. Because of the small sample size constraints, simplifying assumptions about the biological system are used to enable the analysis of ‘omics’ scale data. For example, the differential expression of genes between two conditions may be calculated independently for each gene, even though genes are not independent of each other [41,42]. Despite these simplifications, these analyses can be useful when little is known about the function or the genes targeted by the splicing factors. An alternative approach that has been used extensively is meta-analysis. Meta-analysis is the combination of samples from multiple experiments into one analysis to increase the sample size. In all cases, useful bioinformatics methods applied to high-throughput data will generate hypotheses at a smaller scale, for example the interaction between a few proteins, that can be experimentally validated [43,46].

**Figure 3.**
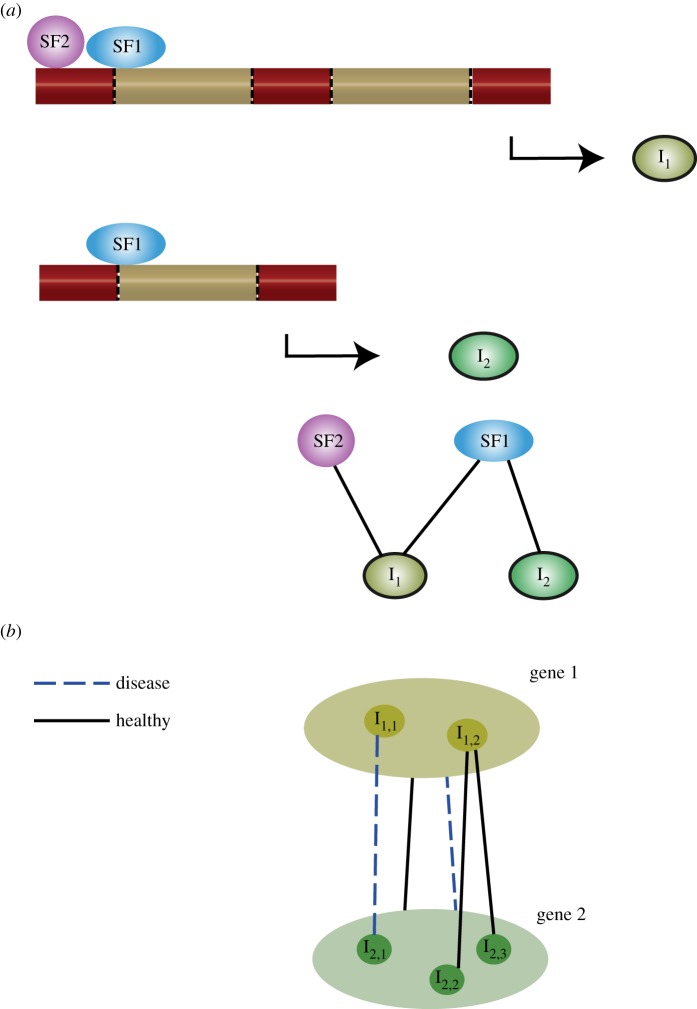
(*a*) Two different isoforms are shown. Isoform 1 (I_1_) is regulated by two splicing factors (SF1 and SF2). By contrast, isoform 2 (I_2_) is only regulated by SF1. The network view of this relationship is shown underneath. (*b*) This example shows a toy network between two genes and their isoforms. At the gene level, a connection between the two genes is shown under both healthy and disease states. However, at the isoform level, differences can be seen in the interaction between isoforms in the healthy and disease states.

We view the modelling of splicing regulation and epigenetics from two, separate but related networks: splicing regulatory networks [47,48] and functional isoform networks [49]. Interactions between the elements responsible for the splicing process are referred to as splicing regulation networks. This includes the *trans*-acting splicing factors and the *cis*-acting binding motifs (figure 3*a*). Epigenetic marks may be viewed in these networks as *cis*-acting motifs that influence the activity of splicing factors [50]. The second type of network is a functional isoform network. In these networks, interactions between different isoforms across genes are modelled. This is used to infer functional units according to the co-expression or shared regulation of isoforms (figure 3*b*). These functional isoform networks are usually specific to either disease status, cell or tissue type [51]. Given the role of epigenetic marks in splicing and disease, epigenetic information can be used to aid the analysis or inference of these networks. Below we discuss examples of resources and models that can be used to perform the analysis of epigenetic and splicing data both at a small scale and using high-throughput data.

## 6. Splicing regulation networks

Splicing regulation networks (SRNs) are used to represent the interactions within and between *trans*-acting RNA-binding proteins (RBPs) or splicing factors and *cis*-regulatory targets or epigenetic marks. In network analysis, the splicing factors and their targets are represented as nodes, while edges between nodes represent that an interaction exists between them. To infer networks, computational methods select the set of nodes and interactions between them that best explain the observed data [52,53]. SRNs can be built for specific disease or tissue types to identify splicing factors that contribute to phenotype. Differential splicing networks can be inferred, reflecting changes in splicing networks under different conditions. Below we provide examples of SRNs that have been inferred and discuss some, though not exhaustive, models from regulatory and signalling network inference that, we believe, can be adapted to model splicing and epigenetics.

A splicing regulatory network for the tissue-specific splicing factor FOX-1 and its paralogue FOX-2 (Fox-1/2) was identified using genome-wide searching for their *cis*-binding motif. The motif for FOX-1/2 was found in exons and intronic flanking regions across the genome. False discovery rates were controlled by using the conservation of motif location in 28 additional vertebrate genomes to categorize FOX-binding targets. RT-PCR was used to validate exon splicing patterns [48]. The work highlighted the combinatorial nature of splicing motifs; differential splicing patterns could not be entirely explained by the FOX-1/2 binding motifs, suggesting a combinatorial regulation in conjunction with additional splicing factors. This opens interesting avenues for future research. Logic models can be used for modelling combinatorial relationship such as OR, AND and NOT between regulators of splicing [37]. For example, if the separate overexpression of two splicing factors led to alternative splicing of the same gene, this would indicate an OR relationship of these two splicing factors and the gene, where either splicing factor can regulate the genes spliced.

To model splicing regulatory networks between *trans*-acting factors using logical relations, the most direct method would be to knock down individual or combinations of splicing factors and measure the expression level of each splicing factor [54,55]. Complete perturbation of the SRN allows for the topology of the network to be inferred. By contrast, any unobserved combinations of splicing factors can lead to uncertainties in resolving the relationships between them. Therefore, this type of analysis requires a known splicing factor or factors to have their expression altered to elucidate targets and function.

To overcome the lack of targeted perturbation data, methods, including Cell Net Optimizer (CellNOptR), have been developed that use prior knowledge networks to facilitate the inference of the logic-based signalling networks. In signalling networks, prior knowledge networks have been used to build initial models of signalling regulation. These initial models are refined using phosphoproteomic data that measure activity in the network following stimulation of the signalling pathway [56,57]. These models can be used to infer SRNs provided the proteins to be included in the model have either been perturbed or their abundance measured across perturbations. In practice, it has been shown that alternative splicing is regulated by signalling pathways, such as MAPK and AKT, through phosphorylation of splicing factors [17]. Therefore, activation of these pathways may be used to provide experimental data for inference. Alternatively, altering histone modifications may provide an indirect perturbation of splicing due to the recognition of histone marks by splicing factors that can be used for modelling. The difficulty with methods such as CellNOptR is having sufficient data to select between different models. Consequently, these methods are often used to resolve interactions between splicing factors but not to identify novel factors contributing to splicing regulation.

RNA-seq data have been used to infer splicing modules and the splicing factors that regulate them. RNA-seq data from multiple experiments in humans were combined to build co-expression networks at an exon level [58]. In this work, a splicing module was defined as a set of co-expressed exons that were assumed to be co-regulated. The analysis of known splicing factor motifs was used to assign splicing factor(s) that regulated each splicing module [58]. These results also showed a combinatorial pattern of splicing factors; multiple splicing modules were enriched for two splicing factor motifs Tra2*α* and SRp30c. Bioinformatics analysis combined multiple experiments that together provided enough data to infer splicing modules. Moreover, ENCODE datasets of transcription factor binding and epigenetic markers were analysed for enrichment within the network, the results of which supported a model of co-transcriptional splicing [59]. While this method identified splicing factors that regulate a set of exons, it did not infer a corresponding network between splicing factors. To do this, methods that have been developed for signalling networks can be adapted to model interactions between splicing factors.

**Figure 4.**
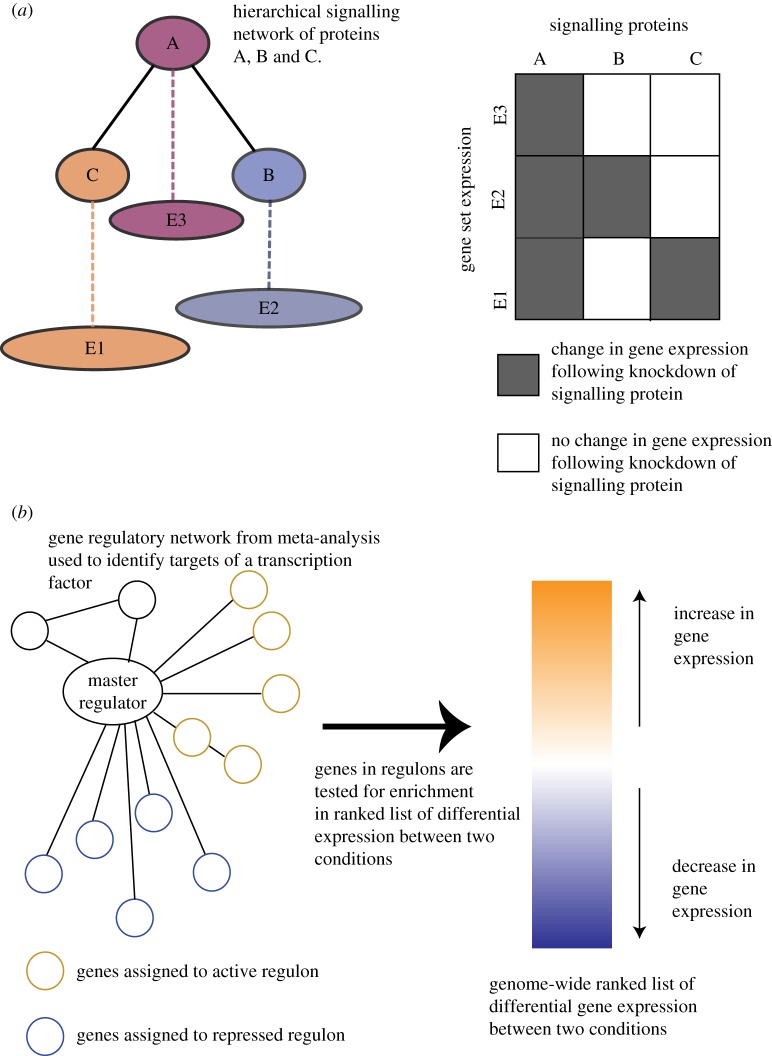
(*a*) The nested effects model uses the effect of knockdowns of signalling proteins (A, B, C), measured using genome-wide expression data, to infer a hierarchical signalling network. In this example, protein A is above B and C in the hierarchy. Knockdown of protein B results in differential gene expression of all genes in gene set E2. Similarly, knockdown of protein C causes differential gene expression of genes in gene set E1. Knockdown of protein A causes differential gene expression in gene sets E1, E2 and E3, as knockdown of protein A changes the expression of proteins B and C. The differences in gene expression following perturbation of the signalling proteins allow a hierarchical network to be inferred. (*b*) In master regulator analysis, first a gene regulatory network (GRN) is inferred based on the co-expression of genes over multiple conditions (meta-analysis). Using the GRN, a transcription factor is assigned two regulons, one containing active genes (brown circles) and the other repressed genes (blue circles). Second, a ranked list of changes in genome-wide expression between two conditions is calculated. For each transcription factor in the GRN, the two regulons are tested for enrichment in the ranked list of genome-wide differential expression. A transcription factor that has the most significantly differentially expressed target genes between the two conditions is hypothesized to be a master regulator of that phenotype.

Nested effects models (NEMs) are an alternative method for inferring signalling networks that can be applied to the analysis of SRNs [60–62]. In contrast to CellNOptR, NEMs use indirect observations of gene expression to infer the signalling network. Using indirect gene expression has the advantage that the data are easier to analyse and a wider repository of publicly available transcriptomic data exists in comparison to proteomic data. The model assumes that the hierarchy of the signalling network can be inferred by using the subset of expression observed at the gene level, following knockdown of the signalling proteins (figure 4*a*). For example, an NEM framework was used to model five genes involved in the invasion pathway in human colon cancer, identifying novel genes involved in invasiveness that were validated experimentally [63].

As with the logic models, the knockdown of splicing factors can be used to infer the hierarchy of effects between a known set of splicing factors using NEMs. However, if the set of splicing factors to be modelled is unknown, an interesting approach would be to use indirect perturbations such as activation of signalling pathways. The effect on splicing of perturbing signalling networks and target splicing factors can be measured by differential exon or isoform expression using RNA-seq data. In the NEM, each node in the network will have a set of differentially spliced genes or differentially expressed exons associated with it. These sets of genes or exons can be searched for common regulatory elements, such as splicing factor motifs and epigenetic marks. Common motifs can also be used to generate hypotheses of elements regulating each of the gene sets [64]. As well as regulatory motifs, epigenetic marks, such as histone modifications, can be integrated with RNA-seq data to elucidate the interactions between epigenetics and splicing.

In humans, integration of multiple datasets has been used to study the relationship between splicing, histone modifications [65] and cytosine methylation [66]. Tissue-independent patterns of DNA cytosine methylation were identified that are related to alternative splicing [66]. By contrast, cell-specific changes in histone modifications were associated with alternative splicing through comparison of RNA-seq, exon arrays and ChIP-seq profiles. Exons displaying cell-specific splicing were found to have less of the H3K36me3 and H3K79me1 histone marks [65]. Thus, bioinformatics analysis of integrated datasets has been successful in identifying relationships between alternative splicing and epigenetic modifications. Master regulators of transcription have also been identified using multiple datasets or metaanalysis [67].

Master regulator analysis (MRA) is designed to discriminate between all regulatory factors to identify a master regulator of a phenotype. The term ‘master regulator’ has been used to describe both transcription factors and splicing factors. Master regulators are defined by their initiation of transcription or splicing networks [68]. In splicing, it is known that RBPs can self-regulate and regulate each other, resulting in single or multiple stable states of the cell [69]. A potential master regulator in splicing, RbFOX2, was identified using iCLIP. The RBP RbFOX2 regulates the autoregulation of several RBPs in mouse embryonic stem cells through alternative splicing-coupled nonsense-mediated decay of the target RBPs [70]. To identify master regulators of transcription, combined multiple experiments (meta-analysis) have been widely used [67].

Meta-analysis relies on the availability of multiple experiments perturbing in different ways a cell line or tissue. Using these combined datasets from multiple experiments, networks of gene regulation patterns were inferred that allowed master regulators of transcription to be identified in prostate cancer [71], breast cancer [72], Parkinson's disease [73] and human B-cells [74]. The networks are inferred by calculating measures of pairwise co-regulation between gene expression; genes involved in the same biological process will show similar patterns of expression over different experimental conditions [74]. Within this gene regulatory network (GRN), transcription factor activity is determined by a two-stage process. First, a transcription factor is assigned a set of active and repressed regulated targets (regulons), based on the genes it is connected to in the GRN. Second, the activity of these targets is used to determine the influence of the transcription factor in the phenotype. Genome-wide differential expression between two conditions is calculated and each gene is ranked according to its differential expression. Each regulon is tested for the enrichment of genes at the top of this ranked list, thus relating the activity of a transcription factor to the contribution of a phenotype via the differential expression of its targets (figure 4*b*) [67]. MRA requires large datasets that perturb the system to generate a large dynamic range of effects in the targets, to successfully identify regulons.

From a splicing perspective, it is possible to perturb the splicing networks and generate high-throughput data that can be used in MRA. Under- and overexpression of RBPs have been used to perturb the splicing system to infer targets and function of alternative splicing [15]. Similarly, drug compounds have been used to reduce methylation to elucidate the impact of epigenetic markers on splicing regulation and disease [75]. This gives mechanisms that can be used to perturb the system that can impact splicing regulation. Previous analysis of GRNs has identified transcription factors rather than splicing factors as central to the network; this is most likely because the underlying network is based on expression at the gene level. Therefore, exon regulatory networks could be used analogously to the GRN to identify master regulators of splicing. Regulatory networks at the exon level have been inferred using RNA-seq datasets, for example for schizophrenia and bipolar disease. This analysis inferred regulatory ‘hubs’ centred around several genes that had specific allelic differential expression between disease and control patients, including CpG islands and splicing enhancers. This analysis required the additional level of information contained within exon expression as the gene-level analysis identified random networks without any gene hubs [76]. Alternatively, splicing motifs can be used to identify splicing factor regulons within the GRN. The differential gene expression profile used for transcription factor MRA can easily be adapted to a differential alternative splicing signature for splicing factor MRA. Furthermore, since epigenetic markers can be assigned to either genes or exons in these networks, this will enable the identification of regulons with shared epigenetic and splicing regulation.

Successful meta-analysis relies on the co-ordination of experiments and outcomes from multiple sources. As a result, databases of experimental data and prior knowledge are required to maximize the data that can be analysed, such as ArrayExpress [77]. The Expression Atlas provides a map of gene expression over different tissues and cell types based on data in ArrayExpress [78,79]. Recently, the ISOexpresso database has been released that gives analogous information but at an isoform level [80]. This includes isoform coverage for different tissue types or disease status. MiasDB contains interactions between splicing factors, epigenetic marks and *cis*-regulatory elements as well as the interaction between *trans*-acting proteins. This resource is currently available only for human interactions and coverage is still incomplete [81]. In future work, there are plans to integrate MiasDB with established signalling pathway database KEGG [82]. It is hoped that this will be extended to allow for ontologies based on splicing regulation. The creation of isoform-specific ontologies will also aid the interpretation of isoform-specific networks discussed in the next section.

## 7. Functional isoform networks

As well as SRNs, networks have also been inferred for the interaction between isoform transcripts [83]. Epigenetic data can be integrated into isoform networks to identify the role of epigenetics in splicing. Understanding the function of changes to transcript expression is fundamental to understanding how different phenotypes materialize. The assumptions underlying these models are that isoforms showing similar patterns of expression share regulators and biological function [84]. The assumption of shared expression equating to shared function has been used extensively in modelling gene regulatory networks. Network views of isoforms provide a more accurate representation than lists of differentially spliced transcripts, where each transcript is treated independently.

SpliceNet built a disease-specific isoform network for lung adenocarcinoma using RNA-seq data. This method identified differences in isoform interactions between *Siva1*, *Cflar* and *Bcl-x* in normal and disease samples not identifiable at a gene level [85]. The gene-level network showed the same dependencies between these three genes in normal and cancer tissues. By contrast, the isoform network showed a cancer-specific relationship between the *Bcl-xL* isoform and a *Siva1* isoform. Literature evidence supports the hypothesis that the binding of SIVA1 to BCL-XL inhibits its anti-apoptotic function [85]. The isoform network, therefore, generated hypotheses of interactions that differ between normal and cancer cells that can be experimentally tested where the gene level analysis could not.

More recently, integration of multiple genomic and proteomic data sources was used to infer a network of functional interactions at the isoform level in mouse [84]. These results found different isoform connections for the two isoforms of *Anxa6* that predicted different functional roles. The connections of one isoform predicted a role in vesicle and organelle fusion, while the second one was connected to isoforms enriched for genes related to the regulation of cell shape. These predictions were supported by experimental evidence in the literature. As the authors note, this is a first step towards building isoform-level networks and future work will focus on building phenotype-, cell- or tissue-specific networks. Moreover, this method integrates heterogeneous data sources including RNA-seq and isoform-docking data, therefore opening the possibility of including epigenetic information in the data used to infer the networks. In this way, interactions between isoforms and epigenetic marks can be identified that are predicted to share a common functional role.

From an epigenetic perspective, the functional isoform networks outlined above can be used as a prior knowledge network and mined using epigenetic data. This is analogous to the work of West *et al.* [86], where age-related gene methylation data were mapped onto a protein–protein interaction network. Modules of age-enriched methylation activity were identified that were associated with stem cell differentiation pathways. In future work, epigenetic markers that are differential with respect to a disease can analogously be mapped to the isoform-specific networks. This would enable identification of isoform-specific functions, not observed at the gene level, that are altered in disease due to differential epigenetic profiles. As well as mining existing signalling or regulatory networks for enriched epigenetic marks, epigenetic marks may also be used to infer the networks.

Epigenetic markers can be used for resolving targets of splicing factors assuming they recognize, or work in combination with, epigenetic marks. As an example, knockdown of PARP1 in *Drosophila* revealed PARP1 to be a ‘splicing hub’ [87]. Nuc-ChIP high-throughput assay was used to find nucleosome targets of PARP1 and alternative splicing was measured through RNA-seq. Experimental validation using ChIP-qPCR showed a reduction of occupancy of PARP1 following knockdown at PARP1-targeted exons, while overall nucleosome density remained constant. A concordant reduction of H3K4me3 was seen at these locations but not after histone H1 knockdown, indicating a specific association between PARP1 and H3K4me3. PAR-CLIP [88] showed that PARP1 facilitates the RNA binding to chromatin, while ChIP pulldown revealed that PARP1 also recruits the splicing factor SF3B1. The multiple mechanisms by which PARP1 influences splicing conferred its role as a splicing hub. The specific interaction between PARP1 and H3K4me3 supports the idea of using epigenetic marks to predict splicing factor binding and regulation. From a modelling perspective, epigenetic marks can be treated as *cis*-regulatory elements to facilitate their integration into isoform networks.

*Cis*-regulatory motif analysis has been used in regulatory networks to identify direct targets of a transcription factor and to distinguish them from downstream effects [55]. Similarly, in co-splicing networks based on the correlation of inclusion of exons, *cis*-regulatory motifs of splicing factors were used to identify splicing factors regulating sets of strongly co-expressed exons [64]. In addition to splicing motifs, integrating epigenetic marks, such as histone modifications and methylation data, can be used to understand the interaction between epigenetic factors and splicing. In the example above, knowledge of the H3K4me3 mark can be used to separate direct targets of PARP1 from indirect effects.

## 8. Current limitations and challenges

The bioinformatics analysis of epigenetics and splicing has so far relied heavily on the meta-analysis of multiple data sources [65,66]. This meta-analysis requires sufficient numbers of experiments from either the same cell type or disease condition that can be combined [85,86]. This restricts the experimental conditions under which epigenetics and splicing can be analysed [76,85]. Furthermore, the annotation of results requires prior knowledge [59,79] but databases on prior knowledge are not complete and exist only for specific organisms [80,81].

Meta-analysis has been successful in identifying both splicing factors and their targets and a combinatorial pattern of splicing factors [64]. However, resolving the interactions between splicing factors has so far been limited. Methods that have been used to infer signalling networks could be used to infer splicing networks [56,60,67]. To understand combinatorial patterns or networks between splicing and *trans*-acting factors, specific experiments will need to be designed [37]. This would involve single and combinatorial knockdowns to investigate the interplay between splicing factors. Similarly, as the tools for differential alternative splicing analysis can now model replicates and complicated experimental designs, targeted splicing factors can be investigated to unravel splicing regulation [89–91]. These experiments should also consider the combined analysis of RNA-seq and ChIP-seq datasets designed to investigate splicing and epigenetic markers.

Although splicing can occur co-transcriptionally, results from gene expression networks indicate that correlation or information-based networks are dominated by transcription as opposed to splicing [67,76]. Correlation of splicing factors and alternative splicing will not be identified by these networks unless splicing results in a concordant decrease in gene expression. Therefore, the analysis of exon co-expression networks [76,85] is expected to increase our understanding of co-transcriptional splicing and give a more complete view of regulatory networks.

Integrating epigenetic data into these exon co-expression networks is a current challenge for bioinformatics. For example, when integrating DNA methylation with gene expression it is common to use the cytosine methylation at promoter regions assuming a negative relationship between DNA cytosine methylation and gene expression [92,93]. However, the relationship between DNA cytosine methylation and splicing is less clear [85,94,95]. The analysis of epigenetic profiles has found a positive association between methylation density and the inclusion of intragenic exons [93]. Therefore, representing epigenetic marks for splicing factor targets that can be used in modelling is a current challenge in bioinformatics [66]. We anticipate future work will model DNA modifications for a gene as several quantities split across different genomic features, such as promoter, exon and intron. The analysis of nonlinear patterns of methylation levels across a gene may provide insight into predictive signatures of phenotypes based on methylation levels.

## 9. Conclusion

Here, we have reviewed recent progress in models that can be used for understanding epigenetic and splicing regulation. Multiple data sources, including gene and protein expression, histone modifications and DNA methylation, can be integrated to give an overall picture of epigenetics, splicing and disease. However, there remain several challenges to fully understanding epigenetics and splicing regulation. This includes selection of appropriate datasets and methods to model epigenetics and splicing. There are multiple methods from modelling signalling or regulatory networks that can be adapted to study and model the effect of epigenetics on splicing. We anticipate that future work will establish how to use genome-wide DNA methylation profiles to create a ‘splicing signature’ that can relate DNA methylation levels or histone modifications to alternative splicing outcomes. While many analyses of single splicing factors have been successful in identifying splicing targets and modes of regulation, we expect future models of splicing to allow for combinatorial mechanisms between splicing factors. Furthermore, we anticipate that recently discovered epigenetic marks, such as adenosine methylation, will be investigated for a potential role in splicing. Identifying the mechanisms by which isoform expression is regulated and its differential expression in diseases will aid understanding of isoform function and provide potential therapeutic targets.

## Data Accessibility

This article has no additional data.
